# A Machine Learning Approach for Real-Time Detection of Inadequate Sedation Using Non-EEG Physiological Signals

**DOI:** 10.3390/bioengineering12101049

**Published:** 2025-09-29

**Authors:** Huiquan Wang, Chunliang Jiang, Guanjun Liu, Jing Yuan, Ming Yu, Xin Ma, Chong Liu, Jingyu Xiao, Guang Zhang

**Affiliations:** 1 School of Control Science and Engineering, Tiangong University, Tianjin 300387, China; huiquan85@126.com (H.W.); chunliang95@foxmail.com (C.J.); maxin2009@tiangong.edu.cn (X.M.); 2 Systems Engineering Institute, Academy of Military Sciences, People’s Liberation Army, Tianjin 300161, China; guan_jun0502@163.com (G.L.); yuanjingjy@163.com (J.Y.); yuming381@sina.com (M.Y.); 3 Department of Anaesthesiology, Tianjin 4th Centre Hospital, The Fourth Center Clinical College of Tianjin Medical University, Tianjin 300140, China; lchong88@163.com; 4 Department of Anesthesiology, Chongqing University Cancer Hospital, Chongqing 400030, China; jyxiao1989@cqu.edu.cn

**Keywords:** inadequate sedation, bispectral index, non-EEG physiological signals, machine learning, out-of-hospital

## Abstract

Sedation is an essential component of the anesthesia process. Inadequate sedation during anesthesia increases the risk of patient discomfort, intraoperative awareness, and psychological trauma. Conventional electroencephalography (EEG) based depth of anesthesia monitoring is often impractical in out-of-hospital settings due to equipment limitations and signal artifacts. Alternative non-EEG-based approaches are therefore required. In this study, we developed a machine learning model to detect inadequate sedation using 27 feature parameters, including demographics, vital signs, and heart rate variability metrics, from the open-access VitalDB database. Patient states were defined as inadequate sedation when the bispectral index (BIS) > 60. We systematically evaluated four temporal windows and four algorithms, and assessed model interpretability using Shapley Additive Explanations (SHAP). The Light Gradient Boosting Machine (LGBM) achieved the best performance, with an area under the receiver operating characteristic curve (AUC) of 0.825 and an accuracy (ACC) of 0.741 using a 2 s time window. Extending the time window to 20 s improved both metrics by approximately 0.012. Feature selection identified 12 key parameters that maintained comparable accuracy, confirming robustness with reduced complexity. These findings demonstrate the feasibility of using non-EEG-based physiological data for real-time detection of inadequate sedation. The developed model is interpretable, resource-efficient, scalable, and shows strong potential for integration into portable monitoring systems in prehospital, emergency, and low-resource surgical settings.

## 1. Introduction

Inadequate sedation during anesthesia poses significant risks, including patient discomfort, intraoperative awareness, and psychological trauma. Timely identification of this state is therefore essential for patient safety, as early detection can reduce adverse outcomes such as pain perception, stress responses, and postoperative complications [1].

In clinical practice, anesthetic depth is typically monitored using physiological parameters such as heart rate (HR), blood pressure (BP), and end-tidal carbon dioxide (ETCO2), in combination with neurophysiological measures, most notably the bispectral index (BIS) derived from electroencephalography (EEG) [2,3,4]. BIS is widely regarded as a reference standard, with values above 60 generally indicating inadequate sedation [5,6]. However, EEG-based monitoring is often infeasible in out-of-hospital or resource-limited environments (e.g., emergency transport, battlefield care) due to high cost, specialized equipment requirements, motion artifacts, and electromagnetic interference [7,8]. These limitations underscore the need for reliable, real-time, non-EEG-based approaches to detect inadequate sedation.

Recent advances in machine learning (ML) and biomedical signal processing have facilitated the use of non-EEG physiological signals such as electrocardiogram (ECG), photoplethysmography (PPG), and heart rate variability (HRV) as surrogate indicators of anesthetic depth. For instance, Chowdhury et al. applied deep learning algorithms to ECG and PPG derived heatmaps, achieving an accuracy (ACC) of 86% in anesthetic state classification [9]. Gang et al. demonstrated the potential of cerebral hemodynamic features as correlates of BIS values [10]. Zhan et al. utilized HRV-derived features with deep neural networks to classify anesthesia depth, while Yin et al. employed long short-term memory models to distinguish consciousness from general anesthesia with high accuracy [11,12]. Moreover, previous studies using the VitalDB database, such as the data-driven investigation of the BIS algorithm by Lee et al., relied on multiple EEG-derived subparameters and complex regression models [7].

Although these studies highlight the feasibility of non-EEG-based monitoring, most efforts have focused on differentiating between deep anesthesia and full consciousness. In contrast, the clinically critical intermediate state of inadequate sedation has received limited attention. This gap is especially concerning in non-operating-room environments, where unrecognized awakening can have severe consequences and EEG based monitoring is often unavailable.

To address this challenge, the present study proposes a machine learning based framework for real-time detection of inadequate sedation using multi-source, non-invasive physiological signals. Leveraging the large-scale VitalDB database, we extracted 27 features including demographics, conventional vital signs, and HRV metrics. Four machine learning classifiers were systematically evaluated across multiple temporal windows, and model interpretability was assessed using Shapley Additive Explanations (SHAP) [13,14,15,16]. This study provides a scalable, interpretable, and resource-efficient solution for sedation monitoring in prehospital, emergency, and other resource-constrained settings.

## 2. Methods

**Figure 1:**
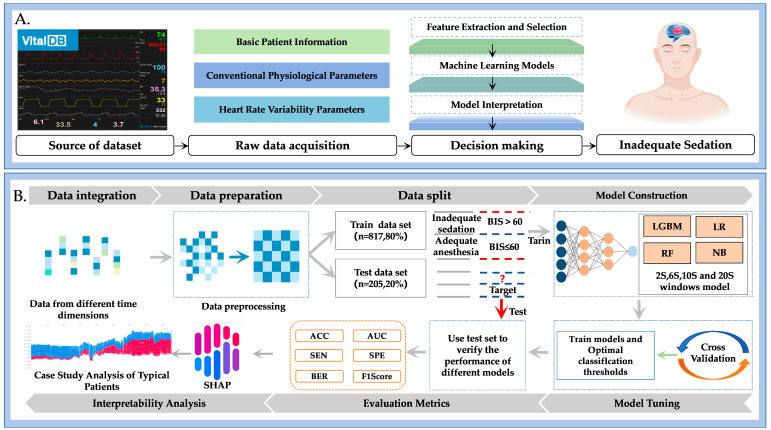
Conceptual framework and workflow of the proposed machine learning approach for detecting inadequate sedation using non-EEG physiological signals. (**A**) shows the conceptual framework comprising four components: source of dataset, raw data acquisition, decision making based on multi-parameter features, and identification of inadequate sedation. (**B**) illustrates the detailed workflow, including data preprocessing, feature extraction, model training and validation, and interpretability analysis using SHAP.

As shown in Figure 1A, the conceptual framework of this study consists of four major components: source of dataset, raw data acquisition, decision making based on multi-parameter features, and identification of inadequate sedation. Building on this framework, Figure 1B further illustrates the detailed workflow for detecting inadequate sedation using non-EEG physiological signals:

1. Data integration: Data from multiple time dimensions and heterogeneous sources are integrated into a unified feature set.
2. Data preprocessing: Signals were denoised, HRV features extracted, missing values imputed, and categorical variables encoded.
3. Data split: Data were split into training (80%) and testing (20%) sets.
4. Model construction: ML classifiers were trained to classify patients as inadequately or adequately sedated using non-EEG features [17,18].
5. Model tuning: Hyperparameters were optimized with 10-fold cross-validation.
6. Evaluation metrics: Models were evaluated on the test set using standard performance measures.
7. Interpretability analysis: SHAP was applied to quantify feature contributions and enhance clinical interpretability.

### 2.1. Source of Data

This retrospective observational study followed the Transparent Reporting of a Multivariable Prediction Model for Individual Prognosis or Diagnosis (TRIPOD) guidelines. Data were obtained from the publicly available VitalDB database (http://vitaldb.net/, accessed on 15 March 2023), which contains high-resolution intraoperative physiological waveforms and clinical records. VitalDB was developed to support artificial intelligence research in perioperative medicine and anesthesia. The database includes 486 intraoperative monitoring parameters, 73 perioperative clinical variables, and 34 time-series laboratory parameters collected from 6388 surgical patients at a single tertiary medical center. Among these, 5543 patients had BIS monitoring records available and were considered for inclusion in this study on inadequate sedation detection [19,20].

### 2.2. Participants and Eligibility Criteria

Based on established clinical literature and to ensure population consistency, this study focused on a relatively healthy adult surgical cohort for developing a machine learning model to detect inadequate sedation [21,22].

Inclusion criteria:

- Age between 18 and 65 years;
- American Society of Anesthesiologists (ASA) physical status classification I–III;
- Body mass index (BMI) > 18 kg/m^2^ and ≤30 kg/m^2^;
- Undergoing elective surgery with a minimum duration of 2 h;
- Receiving total intravenous anesthesia (TIVA) as the primary anesthetic technique.

Exclusion criteria:

- Cranial neurosurgery: Procedures involving the central nervous system were excluded due to potential EEG signal interference and BIS distortion.
- Transplantation or cardiopulmonary bypass: These cases were excluded because of significant and frequent hemodynamic fluctuations that could confound physiological signal interpretation.
- Incomplete data: Patients lacking synchronized ECG, PPG, and BIS waveform data, or exhibiting substantial waveform loss after extraction, were excluded.

**Figure 2:**
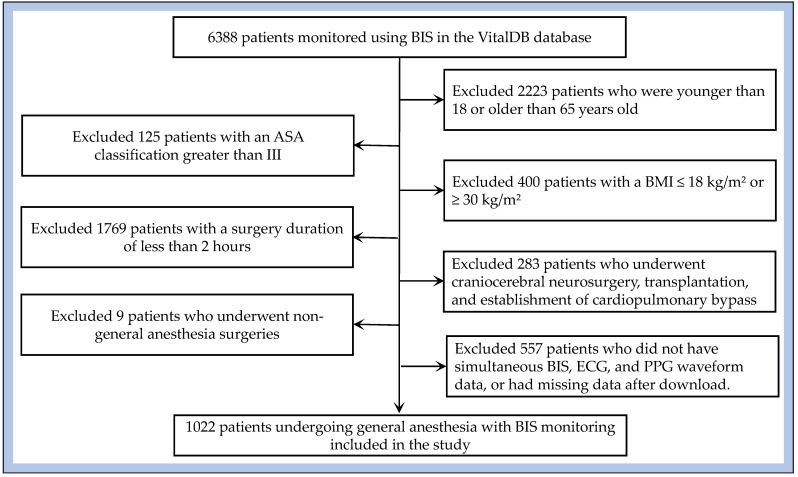
Cohort selection process.

After applying these criteria, 5366 patients were excluded, yielding a final study cohort of 1022 patients. This cohort was used for model training, validation, and analysis. To avoid potential data leakage, train/test splitting was performed at the patient ID level, with 80% of patients allocated to the training set and 20% to the test set. The detailed screening process is illustrated in Figure 2.

### 2.3. Outcome Definition and Predictors

The primary objective of this study was to develop a model for detecting inadequate sedation during anesthesia using non-invasive physiological monitoring data. In clinical practice, BIS is widely used as a surrogate for anesthetic depth, and values above 60 are commonly referenced in guidelines and clinical studies as indicating insufficient sedation, associated with an increased risk of intraoperative awareness, patient movement, or hemodynamic instability [5,6,7,9]. Given the retrospective nature of the dataset, which did not include postoperative interviews to confirm awareness, BIS thresholds were adopted as the operational definition in this study.

Accordingly, binary outcome labels were assigned as follows: inadequate sedation was defined as a BIS value > 60, whereas adequate anesthesia was defined as BIS ≤ 60 [17,18].

This dichotomy reflects a clinically relevant classification consistent with perioperative monitoring practice, particularly valuable in environments where EEG-based systems are not feasible. A total of 27 feature parameters were extracted for analysis (Table 1), with HRV parameter definitions provided in Supplementary Materials (Table S1). These features were derived from the Parameter List of the VitalDB database and accessed through different API views: Clinical Information, Track list, and Data track.

### 2.4. Data Preprocessing and Handling of Missing Data

**Figure 3:**
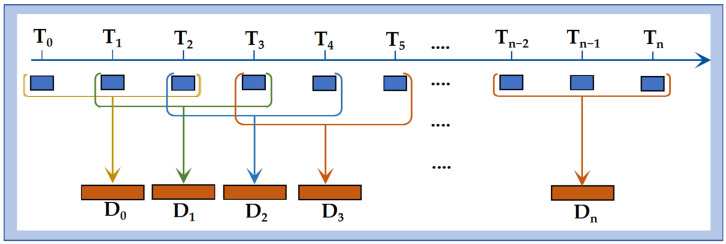
Dynamic time window. Construction of a 6 s sliding window, where T_0_ represents the initial time point for collecting the patient’s physiological data; Tn denotes the sampling moments during the patient’s anesthesia process, with a sampling interval of 2 s; and D_n_ represents the length of the constructed dynamic time window.

Dynamic time window: Physiological parameters inherently exhibit temporal continuity. To capture this dynamic information, a time windowing approach was implemented. The construction of the dynamic window was designed to approximate the calculation principle of BIS, thereby enabling the model to incorporate short-term physiological variability. Detailed definitions and the implementation process are provided in Figure 3.

Heart rate variability calculation: HRV features were computed from 30 s ECG segments after noise reduction and artifact correction, both performed using NeuroKit2, and included time-domain indices, frequency-domain measures, and nonlinear metrics in accordance with established HRV analysis protocols [23].

Missing value handling: The overall missing rate of the extracted physiological monitoring data was less than 10%, ensuring sufficient data completeness. To maintain consistency with real-world clinical monitoring and minimize potential imputation bias, mean imputation was applied only to continuous physiological parameters with missing values. No imputation was necessary for categorical variables, as these were complete in the final cohort. The detailed missing rates for each parameter are provided in the Supplementary Materials (Table S2). Supplementary Materials (Figure S1) presents violin plots of the 23 parameters, illustrating the data after preprocessing.

One-hot encoding of categorical variables: Categorical variables were encoded as binary vectors using one-hot encoding [24]. In this study, gender was the only categorical variable, encoded as 1 for male and 0 for female.

Feature standardization: To ensure comparability across features with different units and scales, all continuous variables were standardized using z-score normalization. Each feature was transformed by subtracting the mean and dividing by the standard deviation, based on the training set distribution [25].

Feature dimensionality across different time windows: Temporal features, defined as conventional physiological parameters and HRV metrics extracted from ECG signals, together with demographic variables, constituted the feature set for model input. The input dimensionality increased with window length: 27 features for 2 s, 142 for 6 s, 234 for 10 s, and 464 for 20 s. Details of the feature composition are provided in the Supplementary Materials (Table S3).

### 2.5. Model Development

Four ML algorithms were tested for real-time identification of patients with inadequate sedation: Random Forest (RF) [26], Light Gradient Boosting Machine (LGBM) [27], Logistic Regression (LR) [28], and Naïve Bayes (NB) [29]. These methods were chosen to represent diverse methodological families, including decision tree ensembles, gradient boosting, regression-based classification, and probabilistic modeling. Model parameters were optimized on the training set using 10-fold cross-validation, with the learning rate adjusted during cross-validation and kept constant throughout the entire training process.

### 2.6. Model Performance and Validation

The performance of the machine learning models was evaluated using multiple standard metrics, including accuracy (ACC), area under the receiver operating characteristic curve (AUC), sensitivity (SEN), specificity (SPE), Bayesian error rate (BER), Matthews correlation coefficient (MCC), F1-score, Cohen’s kappa coefficient (KAPPA), and confidence interval (CI).

### 2.7. Feature Selection Using Recursive Elimination

Recursive feature elimination with cross-validation (RFECV) was used to balance model complexity and predictive performance. Less informative features were iteratively removed to obtain a parsimonious subset of predictors, thereby enhancing both ACC and interpretability. This process reduced dimensionality by eliminating irrelevant or redundant variables without compromising recognition performance [30,31]. The optimal feature subset (OPT_subset) was defined as the configuration yielding the lowest average Bayesian error rate (BER), while the minimum feature subset (MIN_subset) corresponded to the smallest number of features within one standard deviation of the BER in the OPT_subset [32].

### 2.8. Model Explainability Analysis

To enhance interpretability, we applied SHapley Additive exPlanations (SHAP), an explainable AI approach grounded in cooperative game theory, to quantify the marginal contribution of each predictor to the model outputs. SHAP provided consistent and additive attributions and, for the tree-based models used in this study (LGBM), allowed efficient computation through the TreeExplainer framework. The method enabled both global interpretation, by summarizing the average impact of features across the dataset, and local interpretation, by illustrating how specific features influenced predictions for individual patients. From a technical perspective, SHAP offered intuitive visualizations that linked changes in physiological parameters to predicted sedation states. Compared with alternative interpretability techniques, SHAP yielded theoretically consistent attributions with high local fidelity, thereby improving transparency, supporting clinical decision-making, and facilitating potential translation of the model into real-world anesthetic monitoring.

### 2.9. Software and Reproducibility

All data preprocessing, feature selection, model training, and interpretability analyses were conducted in Python 3.9 using open-source packages, including scikit-learn, LGBM, SHAP, pandas, and NumPy. The computational workflow was implemented in a Jupyter Notebook 6.5 environment. Source data were stored in and queried from a PostgreSQL 15.3 database, which enabled efficient data extraction and time-window alignment from the VitalDB relational database. The detailed pseudocode for real-time detection of inadequate sedation using non-EEG signals is presented in the Supplementary Materials (Algorithm S1).

## 3. Results

### 3.1. Baseline Characteristics of Included Patients

The baseline characteristics of the included patients are summarized in the Supplementary Materials (Table S4). No statistically significant differences were observed between the training and test datasets, which were randomly divided. Comparisons between the two datasets were performed using the chi-square test or F-test.

### 3.2. Comparison of Various ML Approaches

**Figure 4:**
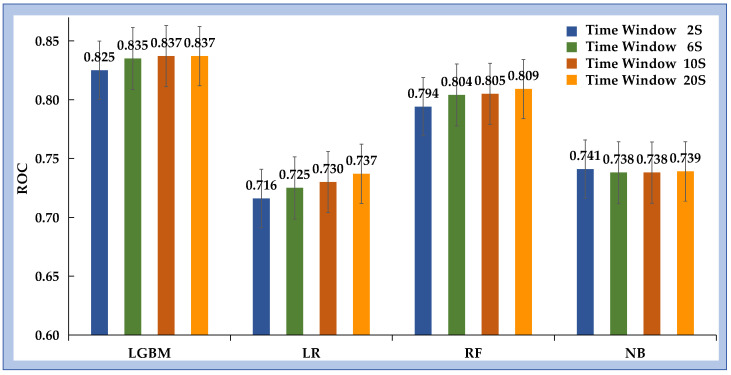
AUC values for the performance of inadequate sedation recognition under different time windows across various ML methods.

Table 2 and Figure 4 summarize the performance of the four ML models across all time windows (2, 6, 10, and 20 s). Among the algorithms evaluated, LGBM consistently achieved the best classification performance, with higher AUC and ACC values than RF, LR, and NB at each window length. Using a 2 s time window, LGBM reached an AUC of 0.825 (95% CI [0.823–0.826]) and an ACC of 0.741 (95% CI [0.740–0.742]), outperforming the other models under identical conditions. From a clinical perspective, minimizing false negatives is critical to avoid unrecognized inadequate sedation. To reflect this requirement, model performance was further evaluated under a fixed sensitivity of 90%. Under this conservative setting, LGBM again outperformed the other algorithms, achieving an ACC of 0.878 (95% CI [0.877–0.878]). The corresponding sensitivity and specificity were 0.900 (95% CI [0.900–0.901]) and 0.595 (95% CI [0.592–0.598]), respectively.

Taken together, these findings demonstrate that LGBM provided the most robust and clinically reliable performance among the evaluated ML approaches.

### 3.3. Analysis of ML Performance Across Different Time Windows

**Figure 5:**
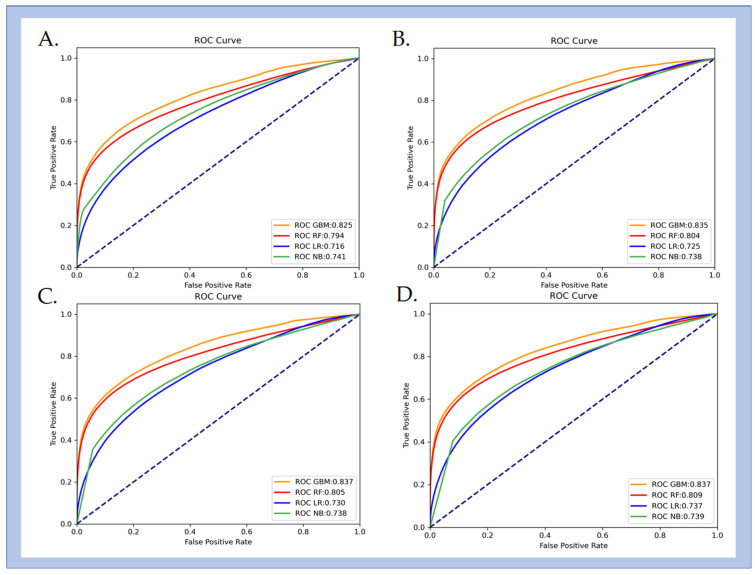
ROC curves illustrating the performance of inadequate sedation identification using different machine learning methods. For inadequate sedation detection at different time windows: (**A**) 2 s, (**B**) 6 s, (**C**) 10 s, and (**D**) 20 s. The dashed diagonal line represents the performance of a random classifier (AUC = 0.5).

To evaluate the effect of temporal resolution, static windows of 2, 6, 10, and 20 s were constructed. For each duration, four machine learning models were trained and assessed using standard metrics. Figure 5 illustrates the AUC trends across different window lengths. The results indicate that LGBM consistently outperformed the other algorithms across all time windows. As the window length increased from 2 to 20 s, the performance of LGBM, RF, and LR improved, whereas NB showed minimal change. LR demonstrated the largest relative improvement, with an AUC increase of 0.021, although it remained inferior to LGBM. Detailed results for each model–window combination are provided in the Supplementary Materials (Table S5).

### 3.4. Analysis of Model Interpretability

**Figure 6:**
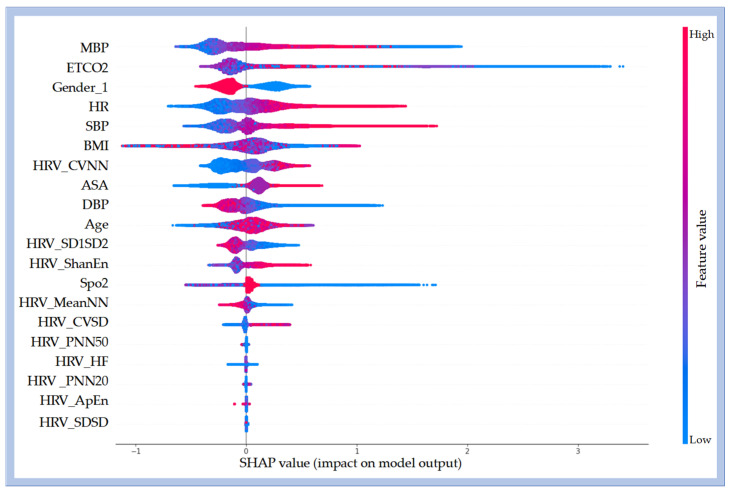
SHAP summary plot illustrates the contribution of input features to the prediction of inadequate sedation based on the LGBM model. The plot shows the top 20 features ranked by their overall importance. Features with greater cumulative impact are positioned higher on the *y*-axis. SHAP values along the *x*-axis represent both the direction and magnitude of each feature’s influence on the model output. Positive SHAP values indicate that the feature increases the predicted probability of being classified into the inadequate sedation group. For continuous variables, the color gradient (red = high values, blue = low values) depicts how changes in feature magnitude affect the classification likelihood.

Model interpretability was assessed using SHAP, applied to the best-performing classifier (LGBM). This method quantified the relative contribution and direction of each physiological and demographic feature to the prediction of inadequate sedation. The analysis identified MBP, ETCO2, SBP, HR, BMI, HRV_CVNN, gender, and ASA physical status as the most influential predictors (Figure 6). Positive SHAP values indicated that higher values of these variables were associated with an increased likelihood of classification into the inadequate sedation group.

Furthermore, the model revealed sex-related differences, with female patients showing a higher likelihood of being classified as inadequately sedated, potentially reflecting physiological differences in anesthetic sensitivity.

### 3.5. The Influence of Feature Selection on the Performance of Algorithms

**Figure 7:**
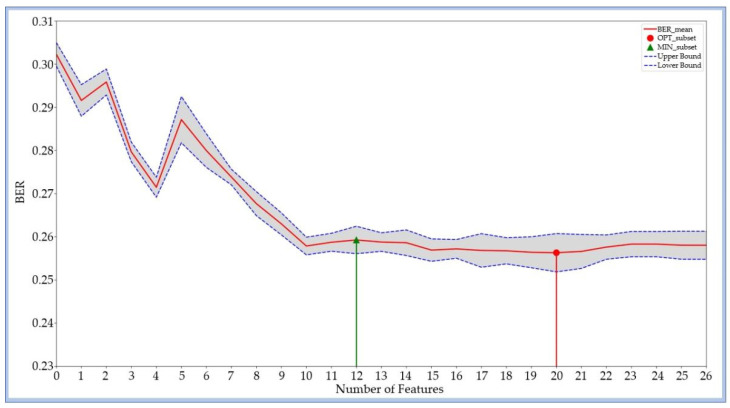
Feature selection curve for the LGBM model in inadequate sedation detection. The *X*-axis shows the number of features included, and the *Y*-axis represents the average balanced error rate (BER) from 10-fold cross-validation. The gray shaded area indicates the standard deviation of BER across folds. Features were added sequentially in descending order of SHAP importance. The red circle marks the OPT_subset (lowest average BER), while the green triangle marks the MIN_subset (fewest features achieving near-optimal performance).

To assess the impact of dimensionality reduction, two feature subsets were derived from the SHAP-based rankings. The OPT_subset included 20 predictors identified as most influential for model performance, while the MIN_subset comprised 12 features that preserved most of the model’s discriminative capacity. Their classification performance is shown in Figure 7 and detailed in the Supplementary Materials (Table S6).

For the LGBM model, the OPT_subset achieved an AUC of 0.827 and an ACC of 0.743, representing a slight improvement over the Sorted full feature set (SOR_subset). The MIN_subset achieved an AUC of 0.825 and an ACC of 0.738 only marginally lower than the SOR_subset, despite a 55.6% reduction in dimensionality.

## 4. Discussion

This study developed a real-time ML model to detect inadequate sedation during general anesthesia using non-invasive, non-EEG physiological signals, with BIS serving as a surrogate reference standard. The results demonstrate that routine intraoperative physiological parameters effectively identify inadequate sedation, particularly in environments where EEG-based methods are impractical, such as prehospital, emergency, and battlefield care. This approach offers a scalable, cost-effective alternative for anesthesia monitoring in resource-limited or mobile clinical settings.

First, among the four ML algorithms evaluated, LGBM exhibited the best performance, achieving an AUC of 0.825 (95% CI: 0.823–0.826) using real-time physiological data. When SEN was fixed at 90%, ACC increased to 0.878 (95% CI: 0.877–0.878). These findings highlight the feasibility of using non-EEG physiological parameters for real-time detection of inadequate sedation, offering a more cost-effective and efficient alternative to traditional BIS systems. In contrast to BIS, which can be expensive and prone to signal interference in high-motion environments, this ML model provides a scalable solution, especially valuable in settings such as emergency care or battlefield care, where rapid decision-making is essential.

Second, the length of the monitoring time window significantly influenced model performance. Extending the time window from 2 s to 20 s resulted in an AUC increase of 0.012, capturing more detailed physiological dynamics and improving prediction reliability. Clinically, longer time windows improve the ACC of detecting inadequate sedation. However, while shorter windows slightly decrease ACC, they offer a faster, more efficient model, which is better suited for high-demand environments requiring rapid decision-making.

Third, the SHAP-based interpretability analysis revealed that several key features (MBP, ETCO2, SBP, HR, BMI, HRV_CVNN, and ASA) significantly impacted the model’s predictions. Increases in MBP, SBP, HR, and changes in HRV_CVNN reflect the body’s physiological response to inadequate sedation, which aligns with clinical observations of inadequate sedation. In Figure 6, the high feature values in red indicate a positive correlation with classification into the inadequate sedation group. The increase in HRV_CVNN corresponds to heightened sympathetic activity. The high importance of ETCO2 reflects the impact of inadequate sedation on respiratory function. Higher BMI may alter the pharmacokinetics of anesthetic drugs, thereby increasing the risk of inadequate sedation, while the ASA classification highlights the challenges of maintaining appropriate sedation in patients with underlying health conditions. The model also predicts a higher probability of inadequate sedation in female patients, which aligns with studies reporting that women exhibit increased emergence sensitivity and faster recovery from anesthesia. These findings emphasize the importance of individualized sedation protocols based on physiological markers and patient demographics, and highlight the model’s potential to optimize sedation management and improve patient safety.

Fourth, this study examined the impact of feature dimensionality on model performance. Initially, the LGBM model utilized 27 physiological features. Reducing the feature set to the top 20 features (OPT_subset) resulted in a slight AUC improvement of 0.002, suggesting that irrelevant or redundant features could introduce noise. Further reducing the model to a minimal set of 12 features (MIN_subset) led to decreases of only 0.002 in AUC and 0.003 in ACC, despite a 55.6% reduction in input dimensionality. These findings highlight the importance of feature selection in simplifying models while maintaining strong classification performance. Clinically, this is particularly significant for real-time or embedded systems in low-resource settings, such as emergency care or battlefield scenarios, where reduced computational complexity and faster response times are critical for ensuring patient safety and facilitating effective decision-making.

**Figure 8:**
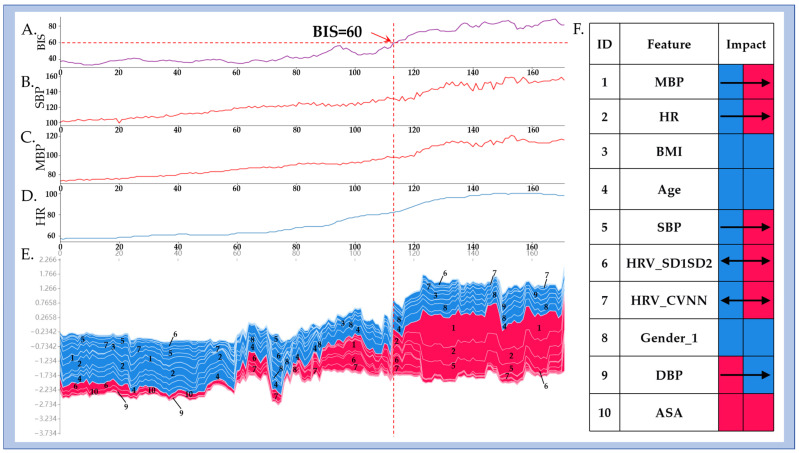
Dynamic influence of physiological parameters on real-time detection of inadequate sedation. (**A**–**D**) show the time series of BIS, SBP, MBP, and HR, with the *x*-axis representing intraoperative time (0–113 points). (**E**) shows the SHAP-based feature contribution map, and (**F**) displays synchronized trends of key physiological parameters.

Finally, to explore the model’s dynamic behavior and interpretability in real-time monitoring, an individual case analysis is presented in Figure 8. This example involves a 64-year-old male patient undergoing general anesthesia for colon cancer surgery, with 172 points, each representing 2 s of continuous intraoperative data. During the initial phase (0 to 113 points), when BIS values remained below 60, the SHAP contribution map (Figure 8E) shows minimal influence of features such as MBP, SBP, and HR on classifying inadequate sedation. However, once BIS exceeds 60, these SHAP regions expand and shift toward red, indicating an increased importance of MBP, SBP, and HR in detecting lighter sedation states.

The consistent trends across Figure 8A–D (physiological time series), Figure 8E (SHAP heatmap), and Figure 8F (aggregated parameter visualization) demonstrate a strong temporal correlation between BIS transitions and model-predicted states. The model is in accordance with the expected physiological changes resulting from insufficient anesthetic drug dosage. As sedation becomes inadequate, sympathetic activation is reflected by elevated HR and BP, which the model accurately predicts. The SHAP-based feature contributions demonstrate the model’s transparency, ensuring it is not a “black box” but a tool offering clear insights into physiological mechanisms driving predictions. This interpretability allows anesthesiologists to trust the model’s decisions, facilitating timely adjustments and improving patient outcomes.

## 5. Challenges, Limitations, and Future Directions

This study has several challenges and limitations that should be acknowledged. First, the dataset was derived from intraoperative cases in a controlled hospital environment, which may not fully represent real-world, out-of-hospital settings such as emergency care or mass casualty incidents. Noise, motion artifacts, and incomplete data in such environments can hinder model performance; therefore, future work should focus on enhancing robustness through denoising algorithms and data augmentation techniques. Second, the study was retrospective and limited to a single center. To improve generalizability and clinical utility, future research will collect real-world out-of-hospital data, expand the sample to include patients of different ages, comorbidities, and anesthetic responses, and conduct multi-center validation. Individualized optimization will also be explored to adapt model parameters to patient-specific characteristics and clinical needs. Third, the reliance on BIS > 60 as the sole definition of inadequate sedation, although clinically accepted, has inherent limitations. BIS is susceptible to artifacts and inter-patient variability, which may introduce label noise into the training process. Future studies should therefore integrate BIS with complementary clinical endpoints such as anesthetic drug concentrations, patient movement responses, and hemodynamic indicators to construct a more precise and robust multimodal ground truth for defining sedation depth. Finally, this approach may offer economic advantages, as models based on routine vital signs could provide a more cost-effective alternative to EEG or BIS based systems in resource-limited settings.

Taken together, these considerations highlight key opportunities for future research, particularly in enhancing robustness, expanding validation, and integrating multimodal endpoints, which will strengthen the methodological foundation and advance clinical translation.

## 6. Conclusions

This study developed and validated a real-time machine learning model that uses non-invasive, non-electroencephalography (EEG) physiological signals to detect inadequate sedation during surgery, demonstrating good performance. By integrating multi-source data, including vital signs and heart rate variability (HRV) metrics, the model offers a cost-effective and scalable alternative to traditional anesthesia depth monitoring systems. This approach is particularly beneficial in high-demand, resource-limited environments, such as emergency medical transport and battlefield care, where out-of-hospital monitoring is required. Future work will focus on further optimizing the model, conducting multi-center validation, and expanding its application to other clinical scenarios to enhance sedation management and improve patient safety.

## 7. Patents

Tiangong University. Huiquan Wang, Chunliang Jiang. et al. Anesthesia depth dynamic identification auxiliary analysis method, device, server and medium: ZL202510458834.5[P] 25 July 2025.

## Figures and Tables

**Table 1:** The 27 feature parameters extracted from the VitalDB database.

Dataset Composition	Detailed Parameters	Data Source
Basic Patient Information	Age, Gender, ASA, BMI	Clinical Information
Conventional Physiological Parameters	HR, DBP, MBP, SBP, SPO2, ETCO2	Data track
Heart Rate Variability Parameters	HRV_Mean, HRV_SDNN, HRV_RMSSD, HRV_SDSD, HRV_CVNN, HRV_HF, HRV_HFn,	Derived from ECG (Track list) after preprocessing
	HRV_pNN50, HRV_pNN20, HRV_LnHF, HRV_SD1,HRV_SD2, HRV_SD1SD2, HRV_S,	
	HRV_ShanEn, HRV_ApEn, HRV_CVSD	

**Table 2:** Results of the 2 s time windows model using various ML methods.

Model	Operating Point	Results (95% CI)							
		AUROC	ACC	SEN	SPE	BER	MCC	F1_Score	KAPPA
LGBM	Sen = Spe	0.825 (0.823–0.826)	0.741 (0.740–0.742)	0.741 (0.740–0.742)	0.741 (0.738–0.744)	0.259 (0.258–0.260)	0.275 (0.273–0.277)	0.294 (0.293–0.296)	0.201 (0.200–0.202)
	Sen of 90%		0.878 (0.877–0.878)	0.900 (0.900–0.901)	0.595 (0.592–0.598)	0.252 (0.251–0.254)	0.376 (0.373–0.378)	0.416 (0.413–0.418)	0.354 (0.352–0.356)
LR	Sen = Spe	0.716 (0.714–0.718)	0.654 (0.653–0.655)	0.654 (0.653–0.655)	0.654 (0.651–0.657)	0.346 (0.345–0.348)	0.166 (0.164–0.168)	0.216 (0.215–0.217)	0.107 (0.106–0.108)
	Sen of 90%		0.862 (0.861–0.862)	0.900 (0.899–0.901)	0.377 (0.374–0.381)	0.361 (0.360–0.363)	0.222 (0.219–0.224)	0.285 (0.282–0.287)	0.213 (0.211–0.216)
RF	Sen = Spe	0.794 (0.793–0.795)	0.703 (0.702–0.704)	0.701 (0.701–0.702)	0.725 (0.722–0.728)	0.287 (0.285–0.288)	0.236 (0.234–0.237)	0.263 (0.261–0.264)	0.163 (0.161–0.164)
	Sen of 90%		0.876 (0.876–0.877)	0.900 (0.900–0.901)	0.565 (0.563–0.567)	0.267 (0.266–0.268)	0.356 (0.354–0.358)	0.399 (0.398–0.402)	0.337 (0.335–0.339)
NB	Sen = Spe	0.741 (0.739–0.743)	0.675 (0.675–0.676)	0.675 (0.674–0.676)	0.675 (0.673–0.679)	0.325 (0.323–0.326)	0.191 (0.190–0.193)	0.233 (0.231–0.234)	0.127 (0.126–0.129)
	Sen of 90%		0.864 (0.864–0.865)	0.900 (0.900–0.901)	0.408 (0.406–0.412)	0.346 (0.344–0.347)	0.245 (0.243–0.248)	0.305 (0.303–0.308)	0.235 (0.233–0.238)

## Data Availability

The data and code used to support the findings of this study are available from the corresponding author on request.

## Supplementary Materials

The following supporting information can be downloaded at: https://www.mdpi.com/article/10.3390/bioengineering12101049/s1, Table S1. Definition of heart rate variability (HRV) parameters. Table S2. Missing Values Table. Table S3. The 27 feature parameters extracted from the VitalDB database. Table S4. Dataset characteristics. Table S5. Results of time window model using various ML methods. Table S6. Outcomes of feature selection for the model: Identification results of the LGBM algorithms for different feature subsets. Figure S1. Violin plots of the 23 physiological parameters included in this study showing the distribution of the parameters after preprocessing. Algorithm S1. Pseudocode for real-time detection of inadequate sedation using non-EEG signals.

## Abbreviations

The following abbreviations are used in this manuscript:

EEG: Electroencephalography; BIS: Bispectral Index; AUC: Area Under the Curve; ACC: Accuracy; SHAP: SHapley Additive exPlanations; LGBM: Light Gradient Boosting Machine; HR: Heart Rate; BP: Blood Pressure; DBP: Diastolic Blood Pressure; MBP: Mean Blood Pressure; SBP: Systolic Blood Pressure; SPO2: Peripheral Oxygen Saturation; ETCO2: End-Tidal Carbon Dioxide; HRV: Heart Rate Variability; ASA: American Society of Anesthesiologists; BMI: Body Mass Index; RF: Random Forest; LR: Logistic Regression; NB: Naive Bayes; ML: Machine Learning; CI: Confidence Interval; ROC: Receiver Operating Characteristic; SEN: Sensitivity; SPE: Specificity; BER: Bayesian Error Rate; MCC: Matthews Correlation Coefficient; KAPPA: Cohen’s Kappa; PPG: Photoplethysmography; ECG: Electrocardiogram; OPT_subset: Optimal Feature Subset; MIN_subset: Minimum Feature Subset; SOR_subset: Sorted Full Feature Subset.
