# Supplementary material for: A Machine Learning Approach for Real-Time Detection of Inadequate Sedation Using Non-EEG Physiological Signals
Source: Bioengineering (Basel). 2025 Sep 29;12(10):1049. doi: 10.3390/bioengineering12101049 (PMC12561100; doi:10.3390/bioengineering12101049)
Supplement: Supplementary file 1 [file bioengineering-12-01049-s001.zip › bioengineering-3864040-supplementary.pdf]

Table S1. Definition of heart rate variability (HRV) parameters.

Number	HRV parameters	Definition
1	HRV_Mean	The mean of the RR intervals.
2	HRV_SDNN	The standard deviation of the RR intervals.
3	HRV_RMSSD	The square root of the mean of the squared successive differences between adjacent RR intervals.
4	HRV_SDSD	The standard deviation of the successive differences between RR intervals.
5	HRV_CVNN	The standard deviation of the RR intervals (SDNN) divided by the mean of the RR intervals (MeanNN).
6	HRV_CVSD	The root mean square of successive differences (RMSSD) divided by the mean of the RR intervals (MeanNN).
7	HRV_pNN50	The proportion of RR intervals greater than 50ms, out of the total number of RR intervals.
8	HRV_pNN20	The proportion of RR intervals greater than 20ms, out of the total number of RR intervals.
9	HRV_HF	The spectral power of high frequencies (by default, 0.15-0.4 Hz).
10	HRV_HFn	The normalized high frequency, obtained by dividing the high frequency power by the sum of low frequency and high frequency powers.

11	HRV_LnHF	The log transformed HF.
12	HRV_SD1	Standard deviation perpendicular to the line of identity. It is an index of short-term RR interval fluctuations, i.e., beat-to-beat variability.
13	HRV_SD2	Standard deviation along the identity line. Index of long-term HRV changes.
14	HRV_SD1SD2	Ratio of SD1 to SD2. Describes the ratio of short term to long term variations in HRV.
15	HRV_S	Area of ellipse described by SD1 and SD2
16	HRV_ShanEn	In HRV analysis, the Shannon entropy (ShanEn) quantifies the uncertainty within the heartbeat interval (RR interval) sequence, with higher values reflecting greater signal complexity.
17	HRV_ApEn	Approximate entropy (ApEn) assesses the regularity and complexity of heart rate signals, providing insight into the randomness and predictability of heart rate fluctuations.

Table S2. Missing Values Table.

Number	Parameter Name	Deletion Rate
1	ETCO2	1.78%
2	DBP	1.48%
3	SBP	1.48%
4	MBP	0.25%
5	HRV_HF	0.08%
6	HRV_HFn	0.08%
7	HRV_LnHF	0.08%
8	HR	0.001%
9	Other parameters	No missing

Table S3. The 27 feature parameters extracted from the VitalDB database.

Time Window	Basic Patient Information	Conventional Physiological Parameters and HRV	Total features
2 s	4	23 × 1	27
6 s	4	23 × 6	142
10 s	4	23 × 10	234
20 s	4	23 × 20	464

Table S4. Dataset characteristics.

	ALL (N=1022)	Train (N=817)	Test (N=205)	P-value
Age	52.123 ± 10.034	52.137 ± 10.118	52.068 ± 9.717	0.253
Height (cm)	164.494 ± 8.272	164.445 ± 8.276	164.685 ± 8.234	0.482
Weight (kg)	62.854 ± 10.230	62.936 ± 10.280	62.527 ±10.050	0.364
Bmi	23.141 ± 2.714	23.183 ± 2.727	22.972 ± 2.660	0.350
Sex				0.483
male	563 (55.1%)	450 (55.1%)	113 (55.1%)	
female	459 (44.9%)	367 (44.9%)	92 (44.9%)	
Asa				0.155
I	318 (31.1%)	246 (30.1%)	72 (35.1%)	
II	615 (60.2%)	501 (61.3%)	114 (55.6%)	
III	89 (8.7%)	70 (8.6%)	19 (9.3%)	

Table S5. Results of time window model using various ML methods.

1. Results of the 2-second time window model using various ML methods.

Model	Operating point	Results (95% CI)							
		AUROC	ACC	SEN	SPE	BER	MCC	F1_Score	KAPPA
LGBM	Sensitivity=Specificity	0.825 (0.823-0.826)	0.741 (0.740-0.742)	0.741 (0.740-0.742)	0.741 (0.738-0.744)	0.259 (0.258-0.260)	0.275 (0.273-0.277)	0.294 (0.293-0.296)	0.201 (0.200-0.202)
	Sensitivity of 90%		0.878 (0.877-0.878)	0.900 (0.900-0.901)	0.595 (0.592-0.598)	0.252 (0.251-0.254)	0.376 (0.373-0.378)	0.416 (0.413-0.418)	0.354 (0.352-0.356)
LR	Sensitivity=Specificity	0.716 (0.714-0.718)	0.654 (0.653-0.655)	0.654 (0.653-0.655)	0.654 (0.651-0.657)	0.346 (0.345-0.348)	0.166 (0.164-0.168)	0.216 (0.215-0.217)	0.107 (0.106-0.108)
	Sensitivity of 90%		0.862 (0.861-0.862)	0.900 (0.899-0.900)	0.377 (0.374-0.381)	0.361 (0.360-0.363)	0.222 (0.219-0.224)	0.285 (0.282-0.287)	0.213 (0.211-0.216)
RF	Sensitivity=Specificity	0.794 (0.793-0.795)	0.703 (0.702-0.704)	0.701 (0.701-0.702)	0.725 (0.722-0.728)	0.287 (0.285-0.288)	0.236 (0.234-0.237)	0.263 (0.261-0.264)	0.163 (0.161-0.164)
	Sensitivity of 90%		0.876 (0.876-0.877)	0.900 (0.900-0.901)	0.565 (0.563-0.567)	0.267 (0.266-0.268)	0.356 (0.354-0.358)	0.399 (0.398-0.402)	0.337 (0.335-0.339)
NB	Sensitivity=Specificity	0.741 (0.739-0.743)	0.675 (0.675-0.676)	0.675 (0.674-0.676)	0.675 (0.673-0.679)	0.325 (0.323-0.326)	0.191 (0.190-0.193)	0.233 (0.231-0.234)	0.127 (0.126-0.129)
	Sensitivity of 90%		0.864 (0.864-0.865)	0.900 (0.900-0.901)	0.408 (0.406-0.412)	0.346 (0.344-0.347)	0.245 (0.243-0.248)	0.305 (0.303-0.308)	0.235 (0.233-0.238)

2. Results of the 6-second time window model using various ML methods.

Model	Operating point	Results (95% CI)							
		AUROC	ACC	SEN	SPE	BER	MCC	F1_Score	KAPPA
LGBM	Sensitivity=Specificity	0.835 (0.834-0.837)	0.750 (0.749-0.750)	0.750 (0.749-0.750)	0.750 (0.747-0.752)	0.250 (0.249-0.252)	0.286 (0.285-0.288)	0.303 (0.301-0.304)	0.212 (0.210-0.213)
	Sensitivity of 90%		0.879 (0.879-0.880)	0.900 (0.900-0.901)	0.610 (0.607-0.613)	0.245 (0.243-0.246)	0.385 (0.383-0.388)	0.423 (0.421-0.425)	0.363 (0.360-0.365)
LR	Sensitivity=Specificity	0.725 (0.724-0.727)	0.661 (0.661-0.662)	0.661 (0.661-0.662)	0.661 (0.659-0.665)	0.339 (0.337-0.340)	0.174 (0.173-0.176)	0.221 (0.220-0.222)	0.114 (0.113-0.115)
	Sensitivity of 90%		0.876 (0.876-0.877)	0.900 (0.899-0.901)	0.389 (0.386-0.392)	0.356 (0.354-0.357)	0.230 (0.227-0.232)	0.292 (0.290-0.294)	0.221 (0.219-0.223)
RF	Sensitivity=Specificity	0.804 (0.802-0.805)	0.740 (0.739-0.740)	0.741 (0.740-0.742)	0.721 (0.718-0.723)	0.269 (0.268-0.271)	0.263 (0.262-0.265)	0.287 (0.285-0.288)	0.193 (0.191-0.194)
	Sensitivity of 90%		0.882 (0.882-0.883)	0.905 (0.905-0.906)	0.579 (0.576-0.582)	0.257 (0.256-0.259)	0.375 (0.373-0.377)	0.416 (0.414-0.419)	0.357 (0.354-0.359)
NB	Sensitivity=Specificity	0.738 (0.736-0.740)	0.675 (0.674-0.676)	0.675 (0.674-0.676)	0.675 (0.672-0.678)	0.325 (0.324-0.327)	0.190 (0.188-0.192)	0.231 (0.230-0.233)	0.126 (0.125-0.128)
	Sensitivity of 90%		0.865 (0.865-0.866)	0.900 (0.900-0.901)	0.431 (0.427-0.433)	0.335 (0.333-0.337)	0.260 (0.258-0.262)	0.318 (0.316-0.320)	0.249 (0.247-0.251)

3. Results of the 10-second time window model using various ML methods.

Model	Operating point	Results (95% CI)							
		AUROC	ACC	SEN	SPE	BER	MCC	F1_Score	KAPPA
LGBM	Sensitivity=Specificity	0.837 (0.836-0.838)	0.751 (0.750-0.751)	0.751 (0.750-0.751)	0.751 (0.748-0.753)	0.249 (0.248-0.251)	0.288 (0.286-0.289)	0.304 (0.302-0.305)	0.213 (0.211-0.214)
	Sensitivity of 90%		0.879 (0.879-0.880)	0.900 (0.900-0.901)	0.613 (0.611-0.616)	0.243 (0.242-0.245)	0.387 (0.385-0.389)	0.424 (0.422-0.426)	0.363 (0.361-0.366)
LR	Sensitivity=Specificity	0.730 (0.728-0.732)	0.664 (0.663-0.665)	0.664 (0.663-0.665)	0.664 (0.662-0.667)	0.336 (0.334-0.337)	0.177 (0.175-0.179)	0.222 (0.221-0.224)	0.115 (0.115-0.117)
	Sensitivity of 90%		0.864 (0.863-0.864)	0.900 (0.899-0.901)	0.395 (0.392-0.398)	0.352 (0.351-0.354)	0.234 (0.232-0.237)	0.295 (0.294-0.297)	0.225 (0.223-0.227)
RF	Sensitivity=Specificity	0.805 (0.803-0.807)	0.744 (0.743-0.744)	0.745 (0.745-0.746)	0.723 (0.721-0.726)	0.266 (0.264-0.267)	0.268 (0.266-0.270)	0.290 (0.288-0.292)	0.197 (0.196-0.197)
	Sensitivity of 90%		0.885 (0.884-0.885)	0.908 (0.908-0.909)	0.581 (0.578-0.584)	0.255 (0.254-0.257)	0.381 (0.378-0.383)	0.422 (0.419-0.442)	0.363 (0.360-0.365)
NB	Sensitivity=Specificity	0.738 (0.736-0.740)	0.677 (0.676-0.678)	0.677 (0.676-0.678)	0.677 (0.674-0.680)	0.323 (0.322-0.325)	0.193 (0.191-0.194)	0.233 (0.231-0.234)	0.128 (0.127-0.130)
	Sensitivity of 90%		0.866 (0.866-0.867)	0.900 (0.900-0.901)	0.433 (0.431-0.436)	0.333 (0.332-0.335)	0.262 (0.260-0.264)	0.319 (0.317-0.322)	0.251 (0.249-0.253)

4. Results of the 20-second time window model using various ML methods.

Model	Operating point	Results (95% CI)							
		AUROC	ACC	SEN	SPE	BER	MCC	F1_Score	KAPPA
LGBM	Sensitivity=Specificity	0.837 (0.836-0.839)	0.753 (0.752-0.754)	0.753 (0.752-0.754)	0.753 (0.750-0.756)	0.247 (0.246-0.248)	0.290 (0.288-0.292)	0.305 (0.303-0.307)	0.215 (0.213-0.217)
	Sensitivity of 90%		0.880 (0.879-0.880)	0.900 (0.899-0.900)	0.616 (0.614-0.619)	0.242 (0.241-0.243)	0.387 (0.385-0.390)	0.424 (0.422-0.426)	0.364 (0.362-0.366)
LR	Sensitivity=Specificity	0.737 (0.735-0.739)	0.671 (0.670-0.672)	0.671 (0.670-0.672)	0.671 (0.668-0.674)	0.329 (0.328-0.331)	0.185 (0.183-0.186)	0.227 (0.225-0.228)	0.122 (0.120-0.123)
	Sensitivity of 90%		0.864 (0.864-0.865)	0.900 (0.899-0.901)	0.405 (0.402-0.408)	0.347 (0.346-0.349)	0.241 (0.239-0.243)	0.301 (0.299-0.303)	0.231 (0.229-0.233)
RF	Sensitivity=Specificity	0.809 (0.808-0.812)	0.751 (0.750-0.752)	0.753 (0.752-0.754)	0.726 (0.723-0.729)	0.260 (0.259-0.262)	0.275 (0.274-0.277)	0.295 (0.294-0.297)	0.204 (0.203-0.206)
	Sensitivity of 90%		0.877 (0.877-0.878)	0.900 (0.899-0.900)	0.598 (0.595-0.602)	0.251 (0.249-0.253)	0.375 (0.372-0.377)	0.413 (0.411-0.415)	0.352 (0.350-0.354)
NB	Sensitivity=Specificity	0.739 (0.737-0.740)	0.681 (0.680-0.682)	0.681 (0.680-0.682)	0.681 (0.677-0.684)	0.319 (0.317-0.321)	0.197 (0.195-0.198)	0.235 (0.233-0.236)	0.132 (0.130-0.133)

Sensitivity of 90%	0.867<br>(0.866-0.867)	0.900<br>(0.899-0.900)	0.440<br>(0.437-0.443)	0.330<br>(0.329-0.332)	0.266<br>(0.264-0.268)	0.322<br>(0.320-0.324)	0.254<br>(0.252-0.257)

Table S6. Outcomes of feature selection for the model Identification results of the LGBM algorithms for different feature subsets.

Feature subset	Result								Number of Features
	AUROC±SD	ACC±SD	Sensitivity±SD	Specificity±SD	MCC±SD	F1_Score±SD	KAPPA±SD	BER±SD	
**MIN_subset**	0.825±0.0025	0.738±0.0062	0.738±0.0070	0.745±0.0058	0.275±0.0037	0.293±0.0038	0.200±0.0047	0.259±0.0021	12
**OPT_subset**	0.827±0.0034	0.743±0.0071	0.743±0.0079	0.744±0.0061	0.278±0.0056	0.297±0.0052	0.204±0.0064	0.256±0.0036	20
**SOR_subset**	0.827±0.0028	0.741±0.0055	0.741±0.0060	0.743±0.0049	0.276±0.0049	0.295±0.0043	0.202±0.0053	0.258±0.0033	27

Minimum Features subset = MIN_subset; Optimum Features subset = OPT_subset; Sorted full feature subset = SOR_subset.

Figures S1

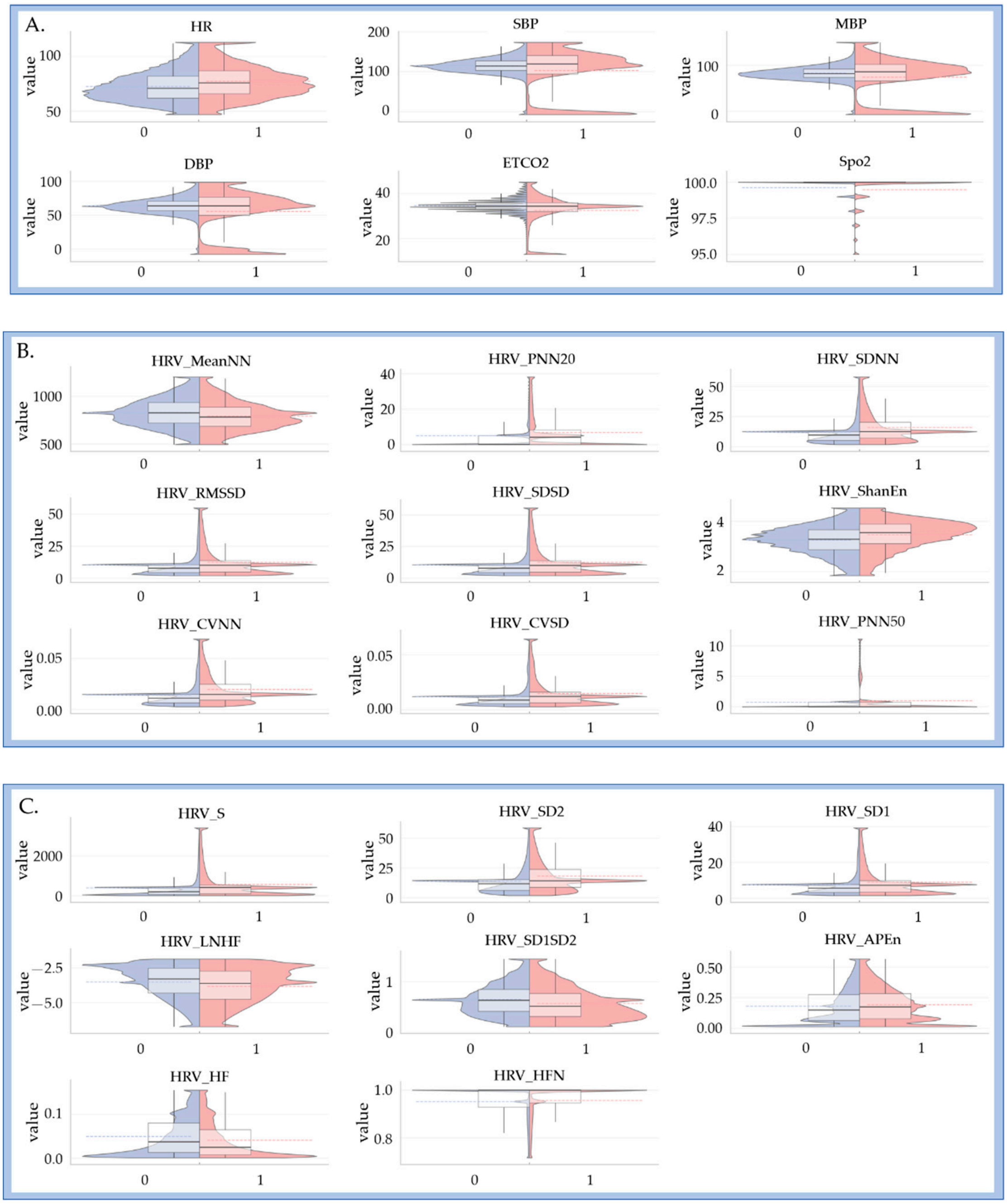

Figure S1. Violin plots of the 23 physiological parameters included in this study, showing the distribution of the parameters after preprocessing.

Algorithm S1.

Pseudocode for real-time detection of inadequate sedation using non-EEG signals.

Inputs:

VitalDB dataset with synchronized ECG, PPG, BIS, demographics, and vital signs

Time windows W ∈ {2s, 6s, 10s, 20s}

Feature list:

Demographics: {Age, Gender, ASA, BMI}, 4 features

Vitals: {HR, DBP, MBP, SBP, SpO2, ETCO2}, 6 signals

HRV metrics (from ECG): 17 metrics

Label rule: Inadequate sedation if BIS > 60, else adequate

Outputs:

Trained classifiers {LR, NB, RF, LGBM}

Performance metrics (ACC, AUC, SEN, SPE, BER, MCC, F1, KAPPA, CI)

SHAP explanations; RFECV feature subsets (OPT_subset, MIN_subset)

Procedure:

1 Load & Screen

1.1 Load VitalDB records; exclude cases per criteria (age, ASA I–III, BMI 18–30, TIVA, duration ≥ 2h).

1.2 Remove cases with poor signal quality or missing synchronized ECG/PPG/BIS.

2 Preprocessing

2.1 Denoise ECG/PPG; correct artifacts (NeuroKit2).

2.2 Compute HRV metrics from 30s ECG segments (time/frequency/nonlinear).

2.3 Align all signals to common timestamps; compute BIS-based labels.

2.4 Handle missing values: mean imputation for continuous variables (<10% missing).

2.5 One-hot encode Gender (binary); keep total base feature count = 27.

3 Dynamic Time-Window Construction

For each W in {2s, 6s, 10s, 20s}:

3.1 Slice continuous streams into sliding windows (step = 2s).

3.2 For each window, assemble feature vector:

X_window = [4 demographics] + [23 non-demo features unfolded over W]

Feature dimension per window:

W=2s: 27

W=6s: 4 + 23×6 = 142

W=10s: 4 + 23×10 = 234

W=20s: 4 + 23×20 = 464

3.3 Standardize continuous features with z-score (fit on train only).

4 Train-Test Split

4.1 Split by patient ID to prevent leakage: 80% train / 20% test.

5 Model Development (per W)

For model in {LR, NB, RF, LGBM}:

5.1 Set class_weight proportional to class frequency (to mitigate imbalance).

5.2 Hyperparameter tuning via 10-fold CV on train set.

- LR: C, penalty

- NB: distributional assumptions

- RF: n_estimators, max_depth, max_features, min_samples_leaf

- LGBM: num_leaves, max_depth, learning_rate, n_estimators, min_child_samples, subsample, colsample_bytree

5.3 Fit model on full train split with best hyperparameters.

6 Evaluation (per W, per model)

6.1 Compute metrics on test set: ACC, AUC, SEN, SPE, BER, MCC, F1, KAPPA; 95% CI via bootstrapping.

6.2 Calibrate threshold at two operating points:

(a) Sen = Spe

(b) Fixed sensitivity target (e.g., SEN ≈ 0.90), report corresponding SPE and ACC.

7 Feature Selection (RFECV on train)

7.1 Run recursive feature elimination with CV using LGBM as estimator.

7.2 Determine OPT_subset (lowest mean BER) and MIN_subset (fewest features within 1 SD of OPT BER).

7.3 Retrain/evaluate models using OPT_subset and MIN_subset; compare performance vs full set.

8 Explainability (on best model, typically LGBM)

8.1 Compute SHAP values (tree explainer).

8.2 Report global importance (top features) and local explanations for representative cases.

9 Reporting

9.1 Summarize per-window performance; highlight trade-off (latency vs accuracy).

9.2 Discuss limitations (BIS label noise, single-center), generalizability, and future work.

End.
